# Monolithic Integration of O-Band InAs Quantum Dot Lasers with Engineered GaAs Virtual Substrate Based on Silicon

**DOI:** 10.3390/nano12152704

**Published:** 2022-08-05

**Authors:** Buqing Xu, Guilei Wang, Yong Du, Yuanhao Miao, Ben Li, Xuewei Zhao, Hongxiao Lin, Jiahan Yu, Jiale Su, Yan Dong, Tianchun Ye, Henry H. Radamson

**Affiliations:** 1 Institute of Microelectronics, Chinese Academy of Sciences, Beijing 100029, China; 2 University of Chinese Academy of Sciences, Beijing 100029, China; 3 Beijing Superstring Academy of Memory Technology, Beijing 100176, China; 4 Research and Development Center of Optoelectronic Hybrid IC, Guangdong Greater Bay Area Institute of Integrated Circuit and System, Guangzhou 510535, China

**Keywords:** Si photonics, InAs/GaAs lasers, epitaxial growth, GaAs virtual substrate

## Abstract

The realization of high-performance Si-based III-V quantum-dot (QD) lasers has long attracted extensive interest in optoelectronic circuits. This manuscript presents InAs/GaAs QD lasers integrated on an advanced GaAs virtual substrate. The GaAs layer was originally grown on Ge as another virtual substrate on Si wafer. No patterned substrate or sophisticated superlattice defect-filtering layer was involved. Thanks to the improved quality of the comprehensively modified GaAs crystal with low defect density, the room temperature emission wavelength of this laser was allocated at 1320 nm, with a threshold current density of 24.4 A/cm^−2^ per layer and a maximum single-facet output power reaching 153 mW at 10 °C. The maximum operation temperature reaches 80 °C. This work provides a feasible and promising proposal for the integration of an efficient O-band laser with a standard Si platform in the near future.

## 1. Introduction

Si-based optoelectronic integration chips (OEICs) are devoted to integrating both electronic devices and photonic devices on mature Si platforms, thus process flows are fully compatible with the standard CMOS technology. On-chip electronic devices are usually the traditional microelectronic devices, such as MOSFETs, TFETs, FinFETs, etc [1,2,3,4]. Moreover, photonic devices mainly include lasers [5,6,7,8], modulators [9,10,11,12], detectors [13,14,15,16,17,18,19], waveguides [20,21,22,23], etc. From the practical perspective, the Si-based on-chip light source was regarded as the final technical hurdle to convert the electron signal into an optical signal, which features the most striking and indispensable unit to achieve high performance Si-based OEICs [24,25,26,27]. To overcome this issue, several strategies were proposed to achieve a high-efficiency on-chip light source. Although group IV-based materials are highly desired to realize the large-scale integrated OEICs, the optical gains for heavily n-type-doped Ge and GeSn are not high enough to overcome the optical loss due to the limited directness and low dimensional material structure growth technique maturity, indicating there is still a long way to go for group IV-based on-chip light sources [28,29,30]. Furthermore, the growth of GeSnSi layers of high quality is also a challenge [31,32]. Benefiting from the connate direct band-gap structure, representative group III–V semiconductor materials, InAs and GaAs, occupy extremely high luminous efficiency [33,34,35,36], making InAs/GaAs quantum dot (QDs) structures directly grown on Si a promising way to develop the high-efficiency on-chip light sources. The main reasons for choosing InAs/GaAs QDs as active optical regions are as follows: outstanding stability, low threshold current density and insensitivity to defects and high temperature, which prompts them to be the ideal choice of light sources used in modern telecommunication networks [37,38,39,40,41]. 

Due to the lattice mismatch, thermal mismatch and polarity difference between Si and GaAs, heterogenous growth facing severe defect problems, such as anti-phase domains (APDs), threading dislocations (TDs) and thermal cracks [42,43,44,45,46,47,48]. These defects act as the non-radiative recombination centers for optoelectronic devices, leading to the aggregation of dopant atoms, triggering the pinning effect, generating additional energy losses and ultimately deteriorating the characteristics of the devices. Although Si-based III–V lasers realized by heterobonding have shown remarkable performance [46,47], III–V lasers fabricated by direct heteroepitaxy on Si and then transferred onto SOI substrate in order to strengthen the gain for OEIC purposes still have unique features in the long run [48,49,50,51,52]. Therefore, crystal quality management for GaAs/Si virtual substrate plays a vital role in solving the technical bottleneck for high performance Si-based InAs/GaAs QD lasers. Up to now, researchers have proposed several methods to grow high-quality GaAs/Si virtual substrates, including thick GaAs buffer layer, thick GaAs/Ge buffer layer, 6° miscut Ge (100), V-groove Si (100) substrate, InGaAs defect-filter layers (DFLs), GaAs/InGaAs superlattice (SLS) buffer layers, patterned Si substrates, AlGaAs seed layers, etc [53,54,55,56]. Through the asymmetric step-graded filter structure, which prompts plastic relaxation, a surface TDD of 2 × 10^6^ cm^−2^ was achieved with a total buffer thickness of 2.55 μm by MBE [55]. Using the approaches described, high performance and high reliability InAs/GaAs QDs laser on Si platforms was demonstrated. In addition, majority buffer layer growth tools are the MBE system, which stands for a low growth rate and high cost, severely limiting its industrial production [50]. To facilitate material growth technology, there is an urgent need to develop the novel growth strategy in order to lower the detrimental impact of electrically active crystalline defects generated from the characteristic difference between Si and III/V materials for high performance InAs/GaAs QDs lasers. Shang C et al. concluded progress in solving these issues, which was regarded as six generations of heteroepitaxial lasers based on Si [57].

Herein, we demonstrate high-quality GaAs/Ge/Si virtual substrate growth via commercially available reduced-pressure chemical vapor deposition (RPCVD) and metal-organic chemical vapor deposition (MOCVD) tools. Detailed Ge RPCVD growth optimization strategies on 8-inch Si are reported elsewhere [58]. To eliminate the APDs defects in the GaAs buffer layer, a three-step growth scheme was introduced (400 °C + 600 °C + 670 °C). When GaAs/Ge/Si virtual substrate growth is performed, molecular beam epitaxy (MBE) was carried out for the deposition of InAs/GaAs QDs structures and an edge-emitting Fabry–Perot (FP) cavity laser with separate confinement heterostructure (SCH) was processed. Characterization methods, such as, high-resolution transmission electron microscopy (HRTEM), atomic force microscopy (AFM), photoluminenscence (PL), light-current-voltage (L-I-V) and continuous-wave (CW) lasing spectrum analysis were implemented. 

The novelty of this study lies in the employment of a 200 nm high-quality GaAs virtual substrate prepared by MOCVD, of which the TDD has been decreased to 7.4 × 10^7^ cm^−2^, with a modified Ge buffer layer that experiences global optimization in each phase during the epitaxy of RPCVD. The state-of-the-art TDD results have reached the 10^6^ cm^−2^ level according to the reported work in ref. [55], but this direct growth scheme with a thin thickness buffer offers a concise and refined solution for competitive InAs/GaAs QD lasers, which saves, layer by layer, SLS structures or substrate patterning and has the future capability to extend to the 12-inch Si wafers that are applicable for surging demands in intra-/inter on-chip data transmission and manipulation.

## 2. Experimental Details

**Figure 1:**
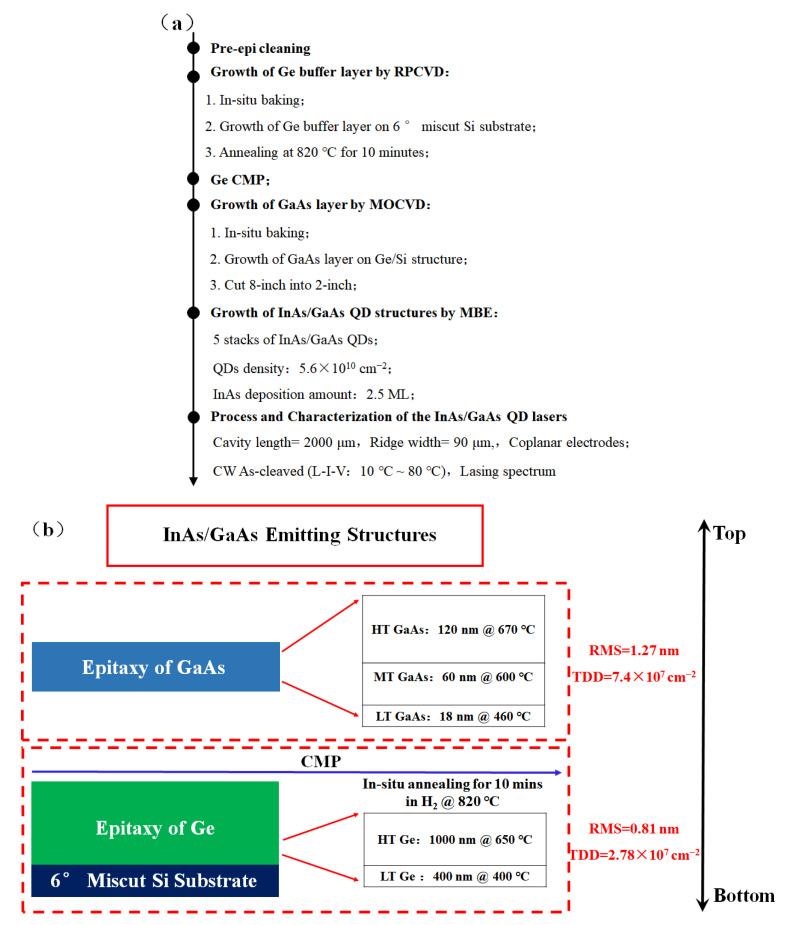
Depiction of the integration of InAs/GaAs QD lasers on Si: (**a**) process flow and (**b**) schematic of the film structure.

Experiments were performed on 8-inch Si (100) wafers with 6° miscut and each sample experienced a standard cleaning procedure prior to epitaxy (SPM followed by APM with DHF last). To avoid any potential contamination on the wafer surface, load-locks were pumped down as soon as they were transferred into equipment. Figure 1a depicts the key process flow in our research and the film stack from bottom to top is vividly shown in Figure 1b. A 1400 nm thick layer of Ge was grown by a two-step growth method in RPCVD (ASM Epsilon 2000, Almere, The Netherlands), including a low-temperature nucleation layer (LT) with a thickness of 400 nm grown at 400 °C and a high-temperature layer (HT) with a thickness of 1000 nm grown at 650 °C. The detailed optimization process flow is illustrated in [58]. As soon as the growth finished, the sample experienced a step of in situ annealing in H_2_ atmosphere at 820 °C for 10 min as well. Threading defect density (TDD) and surface roughness root mean square (RMS) were measured as 2.78 × 10^7^ cm^−2^ and 0.81 nm by HRTEM (Thermo Fisher Talos, Brno, Czech Republic) and AFM (Bruker Dimension Icon Inc., Berlin, Germany), respectively.

Then the surface of Ge was subjected to CMP planarization to offer a flattened basis for the continued GaAs deposition. Like the particulars described in [59], a three-step growth plan was used to fulfill the preparation of ~200 nm thick GaAs by MOCVD (Axitron Crius R, Aachen, Germany). Based on traditional two-step growth, a middle temperature (MT) layer of 60 nm grown at 600 °C was inserted between LT GaAs of 18 nm grown at 460 °C and HT GaAs of 120 nm grown at 670 °C. TDD and RMS were characterized as 7.4 × 10^7^ cm^−2^ and 1.27 nm. Up to this point, we have processed the advanced GaAs virtual substrate based on Si with systematically optimized Ge as the mismatch buffer layer.

**Figure 2:**
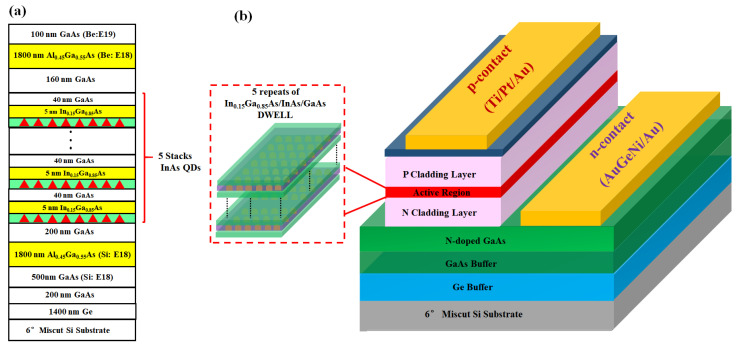
Schematic of: (**a**) the structural growth of InAs/GaAs QDs (**b**) the processed InAs/GaAs QD laser with coplanar electrodes on 6° miscut Si (not to scale).

The schematic of III–V structures is presented in Figure 2a. A 500 nm GaAs buffer layer with a doping concentration of Si: 1 × 10^18^ cm^−3^ was firstly deposited upon the epi-ready virtual substrate to submerge the contaminations and further flatten the GaAs surface. Afterwards, an n-type Al_0.45_Ga_0.55_As layer with a thickness of 1800 nm and same doping concentration was grown at 630 °C to function as the cladding layer. A typical SCH structure was adopted in this laser and 5 stacks of dot-in-well (DWELL) were cyclically deposited, in which each group involves InAs QD layer, In_0.15_Ga_0.85_As capping layer and GaAs spacer layer. The self-organized growth in S-K mode prompts InAs QDs formed in uniform size and dense arrangement. The ambient temperature of QDs growth was 520 °C and the deposition amount was about 2.5 ML (mono-layer) of which the corresponding growth rate was 1.5 ML/minute, affecting the size and morphology of QDs as well as the center wavelength in PL test. InAs QDs in each group act as potential wells in the active region, and the GaAs layer with a thickness of 40 nm plays the role of potential barrier to limit the carrier movement in the QDs. Due to the lattice mismatch as high as 7%, a 5 nm thick In_0.15_Ga_0.85_As layer was introduced for buffering between InAs and GaAs. Waveguides that consist of GaAs and Al_0.45_Ga_0.55_As with a smaller refractive index were constructed on both sides to confine the light field in the active region (top cladding layer). At the top of the laser structure, a highly doped p-type GaAs layer (100 nm with a doping concentration of Be: 1 × 10^18^ cm^−3^) for metal contact was deposited to accomplish the core growth steps.

Followed by several steps as standard lithography, inductively coupled plasma (ICP) etching, SiO_2_ deposition, reactive ion etching (RIE), electrode evaporation, etc. Edge emitting FP InAs/GaAs QD lasers with coplanar electrodes were successfully processed based on the as-grown material as shown schematically in Figure 2b. This Si-based QD laser structure was fabricated into broad-area lasers with a ridge width of 90 µm. As soon as the mesa patternings of metal contacts were opened, the Ti/Pt/Au contact scheme was thermally evaporated onto the p-type GaAs epilayer. In terms of the n-type contact, AuGeNi/Au metallizations were similarly conducted on the exposed n-doped GaAs to form the ohmic contact. After thinning the backside Si to 100 µm, laser bars were cleaved to make a 2000 µm long cavity length. Laser facets were achieved by dicing and polishing where the high-reflection coatings were not applied. The completed devices were afterwards mounted on copper heatsinks with indium solder, and gold wires were simultaneously bonded to enable the smooth measurements of lasing performances.

## 3. Results and Discussion

### 3.1. Material Characterizations

**Figure 3:**
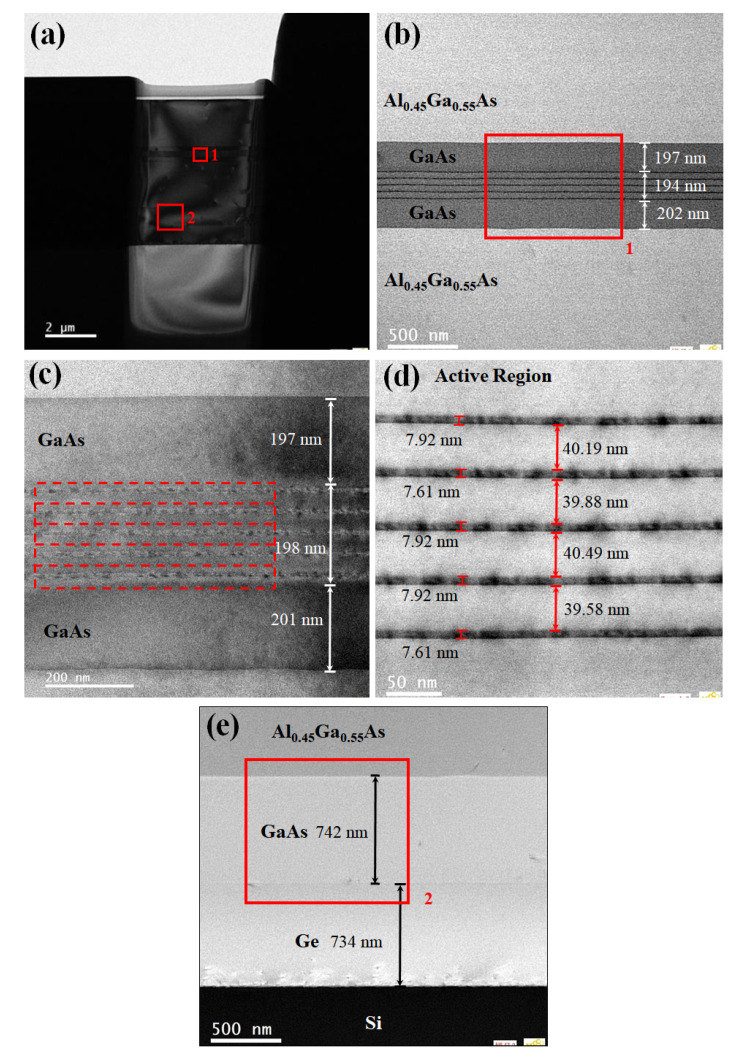
Cross-sectional TEM images of the film stack: (**a**) sample positioning; (**b**) active region; (**c**,**d**) enlarged image of position 1; (**e**) enlarged image of position 2.

Figure 3a displays the positioning of the sample. The area marked in red is enlarged in Figure 3b–e, which is captured around the active region and the engineered GaAs/Ge buffering film. It can be seen that the profile of each stack of InAs/GaAs is clear to distinguish which is evenly distributed, and the overall quality of structure exhibits good quality as revealed. No obvious defects are observed in TEM Figure 3b–d. The high density of the dislocations at the interface between the Si and buffer layers, which originate from the large mismatch in lattice constant and thermal coefficient, have been greatly depleted as the engineering process performed, as is vividly shown in Figure 3e.

**Figure 4:**
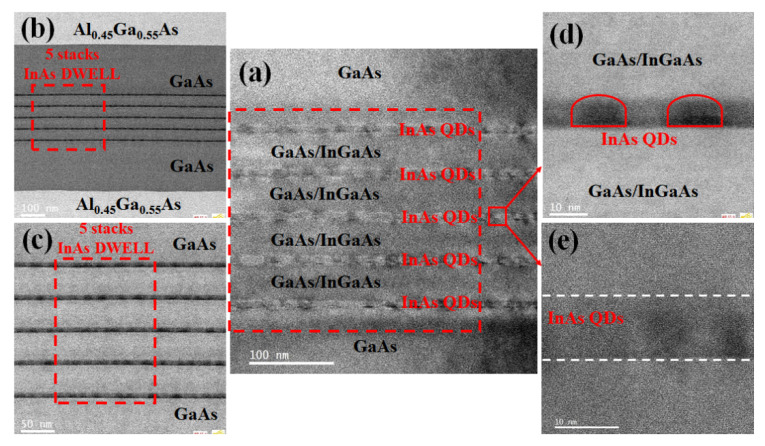
TEM images of the InAs/GaAs active region: (**a**) local active region; (**b**) active region with GaAs structure on both sides; (**c**) magnified image of (**b**); (**d**,**e**) morphology of InAs quantum dots.

As the resolution is further enhanced, the observation results focusing on the InAs/GaAs active region are presented in Figure 4. Figure 4a–c shows the overall morphology of five stacks of cyclic deposition, indicating the distinct boundary of each stack. Dislocation-free or so-called coherent island-like structures prepared by S-K mode have been reported since the 1980s [60]. The S-K growth mode takes advantage of the strain energy generated by lattice mismatch between the materials, and plenty of 3D islands were self-organized with the increase of the material thickness. The wetting layer is formed in a planar mode initially, and 3D accumulation of QD islands on this ultra-thin wetting layer interrupts the 2D planar growth as the critical thickness is exceeded. Figure 4d–e depicts the concrete appearances and crystallinity of the QDs with a regular hemisphere shape and uniform distributions when the local area is magnified. Those QDs are well aligned along the substrate planes, and no defects or Moiré pattern were observed. These results indicate the quantum dots are apparently strained with a high epitaxial quality.

**Figure 5:**
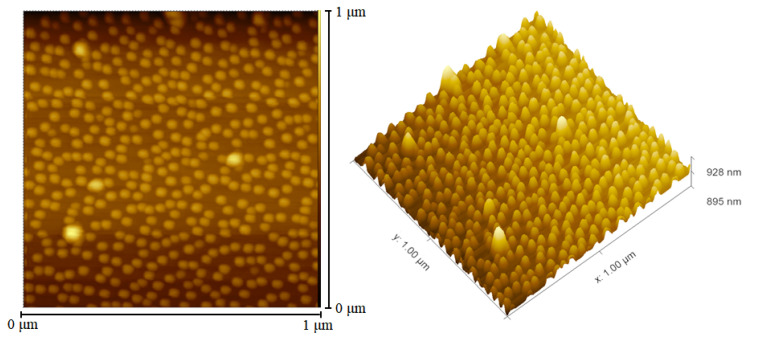
An AFM scanning image of uncapped InAs QDs (1 × 1 μm^2^).

AFM scanning was performed on the surface of the uncapped QD structure (1 × 1 μm^2^), as shown in Figure 5. The performance of QD devices at early stages was often limited by the complexity and instability of the self-organized progress, which led to the fluctuation in the size of the QDs from time to time. This uneven impact caused the broadening of the wavelength variations in the lasing spectrum and the reduction of the peak intensity. Since the size of QDs is comparable to or even smaller than the de Broglie wavelength or the mean free path of electrons in all three dimensions, the movement of electrons in the QDs is significantly limited. Such a confinement effect results in an increase in electron energy and triggers a simultaneous transition from a continuous band to a split structure similar to that of an atom, with quantized energy in three directions. In general, the confinement tends to be more solid on small QDs, which leads to higher electron energy. If the growth temperature is relatively low, the desorption of In atoms decreases and the diffusion length is shortened, which may bring small QDs with high density. However, the atmosphere of low temperature also introduces more defects in the QDs that offset the gain brought by the high density. On the contrary, the density of the islands decreases if the temperature is too high, which promotes the red-shift of the spectrum and the decline of the intensity and even accelerates the serious interdiffusion between the In and Ga atoms. In our experiment, the growth temperature of the InAs QDs was kept at 520 °C, and the growth rate was 1.5 ML/minute. When the surface of the GaAs was gradually covered by small QD islands, a “GaAs pie” sprinkled with “InAs sesame” was obtained. Under this circumstance, the average lateral size of the formed QDs were approximately 30 nm, and the density of QDs was 5.6 × 10^10^ cm^−^^2^ with moderate size and uniform distribution.

**Figure 6:**
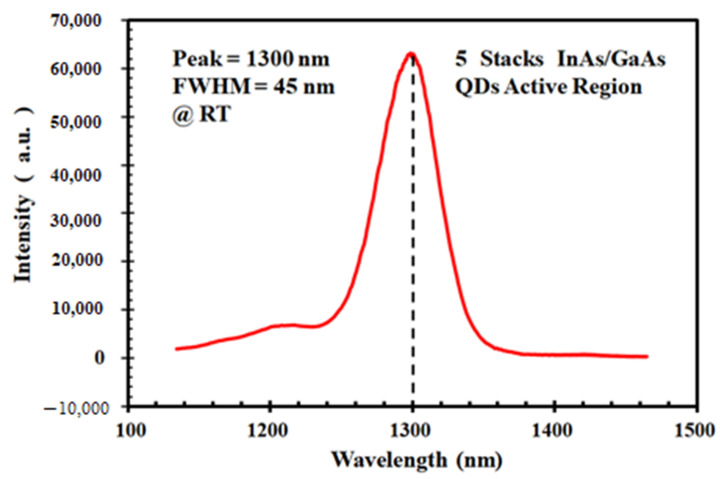
PL result of silicon-based InAs/GaAs quantum dot structure with peak at 1300 nm.

In general, the central emission wavelength for InAs/GaAs locates at the range of 1~1.1 μm. To extend the peak wavelength to 1.3 μm, which is beneficial for lowering the power consumption in optical fiber communication, the method of introducing an In_0.15_Ga_0.85_As buffer layer on the top of the QDs was used to release the strain. Inserting a 5 nm In_0.15_Ga_0.85_As layer of which the lattice constant lies between InAs and GaAs can partially make the strain relax along the growth direction, thereby facilitating a red-shift of the spectrum. Moreover, the non-uniform random diffusion of InAs could be effectively suppressed due to the presence of the In_0.15_Ga_0.85_As buffer layer. As characterized by PL with a liquid nitrogen cooled InGaAs detector, shown in Figure 6, it has a narrow fluorescence property, and the peak wavelength of the luminescence of this structure is exactly located at 1.3 μm (FWHM = 45 nm), which meets the requirements posed by energy-efficient light emitters.

### 3.2. Device Characteristics

**Figure 7:**
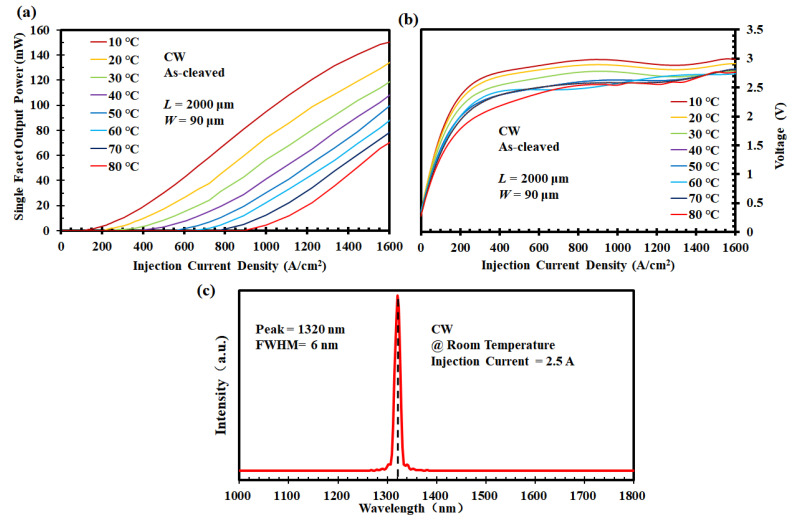
Characteristics of Si-based InAs/GaAs QD laser: L-I-V test (**a**) light output power versus injection current density under different temperatures; (**b**) V-I curves under different temperatures; and (**c**) lasing spectrum of Si-based InAs/GaAs QD laser at room temperature with emission at 1320 nm.

In the realistic scenario of datacom applications where the request of high temperature adaptability is raised, lasers are desirable for proper function in environments with elevated temperatures (up to 80 °C) [61] without the use of thermo-electric cooling. Figure 7 shows the L-I-V characteristic for the broad-area 1.3 µm InAs/GaAs QD laser with a 90 μm ridge width and a 2000 μm cavity length based on the engineered GaAs virtual substrate. Measurements of single-facet output power versus injection current density for our Si-based InAs/GaAs QD laser operated in CW mode from 10 °C to 80 °C are shown in Figure 7a. A threshold current (*I*_th_) of 218 mA was observed at 10 °C, which represents the threshold current density (*J*_th_) of 122 A/cm^2^, and the maximum output power (*P*_out_) of 153 mW was detected without a expression of performance degradation or power roll-off. When it comes to 30 °C, which is basically the same level as the common room temperature, *I*_th_ goes up to 580 mA and *P*_out_ goes down to 126 mW. It depicts the *J*_th_ of 322 A/cm^2^ which corresponds to 64.4 A/cm^2^ for each of the five repeat QD layers. The calculated slope efficiency and external differential quantum efficiency were 0.052 W/A and 5.5%, respectively, which were associated with the facet quality. Moreover, an obvious increasing in *J*_th_ and a rapid decreasing in *P*_out_ were identifiable as the ambient temperature went up. The continuous and stable lasing was still realized even when the atmosphere reached 80 °C, producing output power of 77 mW. Owing to the p-i-n-based laser scheme, the basic characteristics of light-emitting diodes were also embodied and the implementations of device process can be reflected. Process performance, such as doping and contact forming, was examined indirectly through the check of the laser working state. Figure 7b displays similar V-I trends under different temperatures, from which the opening voltage and resistance can be referred. Compared with the PL test, the lasing spectrum that was driven by electric signals with greater energy provides the fundamental features of the device as well. Figure 7c shows the lasing spectrum detected by Fourier-transform infrared spectroscopy, which is measured in an injection current of 2.5 A above the threshold at room temperature. The peak wavelength was confirmed at 1320 nm, which is basically the same as that revealed in the PL spectrum, located in the O-band with a line-width (FWHM) of 6 nm. These characterized results as summarized above (typical L-I-V features, high power output, low threshold current density, slope efficiency and external efficiency) display the prominent lasing behaviors. Furthermore, the 2000 μm cavity length is still too long to observe the multiple longitudinal lasing modes due to the spectrometer limitation, and the related bad cleaving surface for the 6° miscut Si substrate is supposed to be a contributor as well.

**Figure 8:**
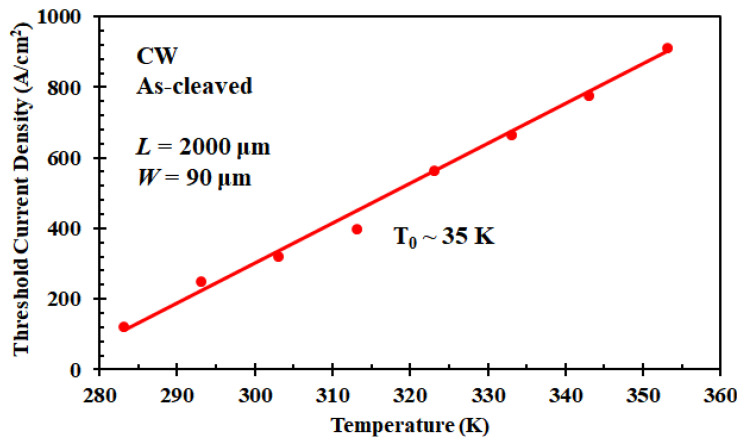
Characteristics of the temperature-dependent threshold current density of Si-based InAs/GaAs QD laser (characteristic temperature).

The sensitivity of the device to temperature variations is negatively correlated with the characteristic temperature *T*_0_. As shown in Figure 8, the temperature dependence of the threshold current density for the packaged laser was recorded and plotted, which follows the exponential function of *I*_th_ ∝ exp (*T*/*T*_0_), as illustrated in Equations (1) and (2). *T*_0_ was deduced as 35 K, which is a typical value for the active region without doping [62,63], by fitting the curve in the range of 10 °C to 80 °C. Generally speaking, this not-so-good feature in the 1300 nm InAs/GaAs QD laser comes from the hole excitation out of the lasing state [64,65]. It is also worth emphasizing that here *T*_0_ was extracted under CW input mode, which underestimates performance as a result of the unavoidable self-heating effects.

(1) $$I_{\mathrm{th}}=I_{0}e^{\frac{T}{T_{0}}}$$

(2) $$T_{0}={\left(\frac{\mathrm{dIn}I_{\mathrm{th}}}{dT}\right)}^{-1}$$

Table 1 presents the recent efforts dedicated to the development of O-band Fabry–Perot lasers with InAs/GaAs QD active regions. Crucial material structures and device parameters are summarized, including adopted substrates, defect buffering layers, emission wavelengths, threshold current density and maximum output power. Progress in different aspects has been made to varying extents. Although the device performances in this work are not advantageous in all aspects, they still show great competence in terms of the listed parameters among typical reported lasers without high-reflection coatings as well as the relative concise growth procedure benefited by the engineered GaAs virtual substrate based on the comprehensively optimized Ge/Si in which the TDD was successfully reduced to 7.4 × 10^7^ cm^−2^. Since the direct growth of GaAs material on unpatterned miscut Si eliminated the complicated design of the defect filtering layer (DFL) and remaining competitive properties, there exists a promising application of this epitaxial plan that alleviates the expensive life-cycle cost.

##  4. Conclusions

We have investigated monolithically integrated InAs/GaAs QD lasers on Si substrates with high-quality GaAs and Ge materials as buffer layers. Room temperature CW lasing at 1320 nm was observed, with a minimum threshold current density of 122 A/cm^2^ and a maximum output power of 153 mW at 10 °C. As the ambient temperature rose to 80 °C, stable continuous lasing was still observed and the output power decreased to 77 mW. In contrast with other schemes, the effective growth applied in this work exploited the comprehensive optimization of the multi-step CVD deposition of GaAs and Ge interlayers with reduced TDD of 7.4 × 10^7^ cm^−2^, which omits the introduction of intricate defect depletion designs. Devices fabricated through this direct growth procedure exhibit performance that compares favorably with reported O-band QD lasers based on Si. We believe a practicable and perspective strategy of the monolithic integration of InAs/GaAs QD lasers with Si, emitting exactly in the O-band, is provided, and it enjoys advantageous material quality as well as favorable device characteristics.

## Figures and Tables

**Table 1:** Benchmarking of recent progress made in O-band InAs/GaAs QD lasers monolithically integrated on Si.

Year	Substrate	Interlayers	TDD (/cm^2^)	Groups of QDs DWELL	Size	Performance					Refs.
					Cavity Length (μm) × Ridge Width (μm)	Operation Condition	λ (nm)	*J*_th_ (A/cm^2^)	*P*_out_ (mW)	*T*_0_ (K)	
2011	6° miscut Ge (100)	1500 nm GaAs	1 × 10^4^ ~ 1 × 10^6^	5	5000 × 50	RT-CW As cleaved	1305	55.2	28	40	[66]
2012	6° miscut Si (100)	2000 nm GaAs/ 2000 nm Ge	5 × 10^6^	5	3500 × 20	RT-CW As cleaved	1280	64	93	37	[6]
2014	6° miscut Si (100)	2000 nm GaAs/ 500 nm Ge	10^8^	7	937 × 4	RT-CW Coated	1250	426	176	100 ~ 200	[67]
2016	4° miscut Si (001)	[InGaAs/GaAs] SLS/ 1000 nm GaAs/6 nm AlAs	10^5^	5	3200 × 50	RT-CW As cleaved	1315	62.5	105	51	[62]
2017	V-Groove Si (001)	760 nm GaAs/ [InGaAs/GaAs] SLS/ 1080 nm GaAs	7 × 10^7^	5	1200 × 10	RT-CW Coated	1250	607	84	NA	[63]
2017	GaP/Si (001)	3000 nm GaAs (with InGaAs DFL)	7.3 × 10^6^	4	2600 × 8	RT-CW Coated	1285	132	175	32	[68]
2017	On axis Si (001)	[InGaAs/GaAs] SLS/400 nm GaAs	NA	5	3000 × 25	RT-Pulsed/ RT-CW	1292	250/425	130/ 43	32	[69]
2018	On axis Si (001)	3100 nm GaAs (with InGaAs DFL)/45 nm GaP	8.4 × 10^6^	5	1341 × 2.5	20 °C-CW Coated	1299	198	185	NA	[70]
2018	On axis Si (001)	[InGaAs/GaAs] SLS/800 nm GaAs/ 40 nm AlGaAs	3 × 10^7^	8	2000 × 80	RT-Pulsed As cleaved	1250	320	30	51	[71]
2019	On axis Si (001)	[InGaAs/GaAs] SLS/800 nm GaAs/40 nm AlGaAs	4.7 × 10^7^	8	1100 × 7	20 °C-CW As cleaved	1225	370	53.2 mW/A	50	[72]
2019	V-Groove Si (001)	[InGaAs/GaAs] SLS/300 nm GaAs/200 nm InGaAs/1600 nm GaAs	3 × 10^6^	5	1450 × 10	RT-CW Coated	1280	286	75	30 ~ 35	[73]
2019	On axis Si (001)	[InGaAs/GaAs] SLS/700 nm GaAs	NA	5	3000 × 50	RT-CW As cleaved	1330	160	48	60.8	[74]
2020	Quasi-nominal Si (001)	[InGaAs/GaAs] SLS/1100 nm GaAs	3 × 10^7^	5	1270 × 6	RT-CW As cleaved	1270	173	52	41	[75]
2020	4° miscut Si (001)	[InGaAs/ GaAs] SLS/350 nm GaAs/300 nm Ge	4 × 10^6^	7	3000 × 25	RT-Pulsed As cleaved	1280	200	78	153	[76]
2021	On axis Si (001)	InGaAs asymmetric graded filter/45 nm GaP	1.5 × 10^6^	5	1500 × 5	RT-Pulsed As cleaved	~1300	266	65	167	[77]
2022	6° miscut Si (100)	200 nm GaAs/ 1400 nm Ge	7.4 × 10^7^	5	2000 × 90	10 °C-CW/ 30 °C-CW As cleaved	1320	122 (10 °C) /322 (30 °C)	153 (10 °C) /126 (30 °C)	35	This work

## Data Availability

The data is available on reasonable request from the corresponding author.
